# Supplementary material for: Chromothripsis during telomere crisis is independent of NHEJ, and consistent with a replicative origin
Source: Genome Res. 2019 May;29(5):737–49. doi: 10.1101/gr.240705.118 (PMC6499312; doi:10.1101/gr.240705.118)
Supplement: Supplemental Material [file supp_gr.240705.118_Supplemental_file_1.zip › contigs/annotated_contigs/DB51/contig.2.DB51_length_355_mean_cov_7.53802816901.docx]

**DB51_length_355_mean_cov_7.53802816901**

```
TGAGGTTTTCACCAATTCCAAACATGAGTAGGAACCAAAAGTAATTAGGCTAAGAACACAGCCAAAGACATGGCACAGGAAAAGCCTAC
 >chr1:33511096-33511220 - E=3e-63
CTGGACAGCCACCACTGGCCACGGTCCA|CCCACTG|CTGTCAAAGCGTGTCACTCGGCCTTCAAGCCCTTCCCAGATCTAGCCCTGCC
                             >chr1:33583941-33584179 - E=5e-132
TCCCTCTGCAGTCACAGCCACTCTCCATGCGAGCCTTGAATGCACCACACTTGCTCTCCGTTCCTTTCTTCTGGGAAGTCCTACCACCC

ACTCCTGTAGTTGACTCCCACTTCCTCAGGGAGGCCTGGGCCTCAGGGAGGGCTCTGCACTTCCACCAGGCACAGTATTATCACAAGGG

C
```
